# Impact of Drying Conditions on Antioxidant Activity of Red Clover (*Trifolium pratense*), Sweet Violet (*Viola odorata*) and Elderberry Flowers (*Sambucus nigra*)

**DOI:** 10.3390/ma15093317

**Published:** 2022-05-05

**Authors:** Agnieszka Zawiślak, Renata Francik, Sławomir Francik, Adrian Knapczyk

**Affiliations:** 1 Department of Biotechnology and General Technology of Food, Faculty of Food Technology, University of Agriculture in Krakow, Al. Mickiewicza 21, 31-120 Kraków, Poland; 2 Department of Bioorganic Chemistry, Chair of Organic Chemistry, Jagiellonian University Medical College, Medyczna 9, 30-688 Kraków, Poland; renata.francik@uj.edu.pl; 3 Institute of Health, State Higher Vocational School, Staszica 1, 33-300 Nowy Sącz, Poland; 4 Department of Mechanical Engineering and Agrophysics, Faculty of Production Engineering and Energetics, University of Agriculture in Krakow, Al. Mickiewicza 21, 31-120 Kraków, Poland; slawomir.francik@urk.edu.pl (S.F.); adrian.knapczyk@urk.edu.pl (A.K.)

**Keywords:** edible flowers, antioxidant activity, polyphenols, anthocyanins

## Abstract

Flowers of red clover (*Trifolium pratense*), sweet violet (*Viola odorata*) and elderflowers (*Sambucus nigra*) were dried by means of air drying at 30 °C and 50 °C and by freeze drying. The content of polyphenols was determined using the Folin–Ciocalteu reagent, while anthocyanins were quantified by the pH differential method. Antioxidant activities of aqueous and ethanolic extracts of the dried flowers were measured by the DPPH and ABTS assays, as well as FRAP and reducing power methods. The highest amount of polyphenols was determined in the ethanolic extracts of fresh red clover flowers (854.76 mg/100 g), while the highest concentration of anthocyanins was determined in the aqueous extracts of fresh sweet violet flowers (99.41 mg/100 g). The results showed that, in general, the extracts of red clover flower were characterized by the highest antioxidant activity, while the sweet violet extracts had the poorest antioxidant properties, although these values fluctuated depending on the method used. There was strong correlation between antioxidant activity and TPC (r = 0.9196, FRAP method). In most cases, freeze drying was found to be the best conservation method, retaining well the antioxidant properties of the tested flowers and the compounds determining these properties.

## 1. Introduction

Edible flowers have been utilized in traditional medicine as well as cuisine in various regions of the world. They are used as ingredients for salads, desserts, beverages and tea blends. Edible flowers are a rich source of bioactive substances, such as polyphenols and anthocyanins. These compounds are characterized by good antioxidant properties, which makes the dishes gain new, higher nutritional quality. Bioactive compounds contained in edible flowers can protect humans against many chronic diseases, as well as the food itself against oxidative changes. Besides enriching meals with valuable ingredients, edible flowers enhance the appearance and aroma of food preparations, which also stimulates the consumer to try such products [1,2,3,4,5].

So far, there is no formal catalog of edible and non-edible flowers [6].

The genus *Trifolium* from *Fabaceae* family includes about 240 species of clover [7]. Currently, the therapeutic use of *Trifolium* plants is still mainly set upon the recommendations of traditional medicine, but the number of scientific reports documenting their medical effects is increasing. Clover is a rich source of isoflavones, which have curative effects in diseases associated with hormonal disorders and inflammations [8]. *T. pratense* has been used by various cultures to treat eczema and psoriasis. There are data from prostate cancer studies indicating anticancer properties of red clover [9]. Some of the *Trifolium* species may show antioxidant properties, probably due to the presence of many phenolic compounds, such as phenolic acids, catechins, saponins and others [10].

Sweet violet (*Viola odorata*) coming from mountainous part of northern India [11] has been very popular in cosmetology throughout centuries because of its very characteristic sweet scent. Despite the fascinating aroma, *Viola odorata* has been reported to have, among others, antibacterial, anti-inflammatory, antipyretic, diuretic, anticancer and antioxidant properties. These traits have made this herb traditionally used in Ayurvedic and Iranian medicine, as well as in the Lenape culture. Sweet violet is used in treating kidney and liver disorders, whooping cough, asthma, tonsillitis, diabetes and cancer. It is very efficient in treating headache and migraine. The healing properties of *Viola odorata* result from the presence of bioactive compounds, such as cyclotides, flavonoids, alkaloids and triterpenoids [11,12,13].

Elderberry (*Sambucus nigra*) is a deciduous shrub or small tree belonging to the *Adoxaceae* family. It is a widespread, easy-to-grow plant with fruits and inflorescences that have been commonly used in folk medicine. Elderflowers are rich in bioactive substances, mainly polyphenols, from which the most crucial are hydroxycinnamic acids, flavonols and flavanones. Due to the presence of these compounds, flowers of *Sambucus nigra* show antipyretic, diaphoretic, diuretic ant antibacterial properties [14]. It was also reported that substances found in elderflowers can have an antidiabetic effect. In addition to medical applications, elderberry flowers are used as an addition to beverages and desserts due to their unique aroma [15,16].

Edible flowers are highly perishable and should therefore be preserved accordingly. Today, edible flowers are sold fresh in small plastic holders, refrigerated or dried. They are also conserved in sugar and in form of distillates. Appreciating the potential of edible flowers, the food industry continues to search for the best preservation methods for this delicate material [3].

The aim of this work was to examine the influence of different drying conditions on the antioxidant properties and on compounds determining these properties in three species of edible flowers, i.e., elderflowers (*Sambucus nigra*), fragrant violet (*Viola odorata*) and red clover (*Trifolium pratense*). Moreover, two most popular, from a consumer’s point of view, solvents were tested to find the one with better selectivity toward bioactive compounds. Selecting an appropriate solvent is crucial to obtain an extract with appropriate characteristics [17]. The solvents used in this study are suitable for food and pharmaceutical applications, so they can be used in the development of new products based on dried edible flowers. This work is an introductory phase to a more comprehensive study into the possibility of creating products based on the examined edible flowers.

## 2. Materials and Methods

### 2.1. Material Preparation

Flowers of *Viola odorata*, *Trifolium pratense* and *Sambucus nigra* were collected in Bielańsko—Tyniecki Landscape Park in Kraków during spring 2019. The soils in this area are classified as heavy and very heavy [18].

The inflorescences of each species were separated without preliminary washing and divided into four parts. One part of each material was taken fresh for analyses. The remaining parts of the material were dried in different conditions. The methods used were: freeze drying (Labor, MIM OE 950; Budapest, Hungary), air drying at 30 °C (Stöckli, 76.74; Netstal, Swizerland) for 5 h and air drying at 50 °C (Stöckli, 76.74; Netstal, Swizerland) for 3 h.

The dry matter content both in fresh and dried material was determined by the oven method at 105 °C [19].

#### 2.1.1. Ethanolic Extracts Preparation

To prepare ethanolic extracts, 5 g of fresh flower material was subjected to extraction with ethanol and 2% hydrochloric acid solution (95:5 *v*/*v*, 95 mL) by homogenization at 25 °C for 5 min. Then, the homogenate was stirred for 45 min and centrifuged at 5500 rpm for 10 min. The obtained supernatant was decanted from the sludge to give a 5% ethanolic extract, which was intended for analyses. In the case of dried flower samples, the same procedure was applied. However, the weight of dried flower samples was recalculated, based on dry matter, to obtain 5% ethanolic extracts, imitating extracts from fresh raw material.

#### 2.1.2. Preparation of Infusions

To prepare infusions, 5 g of fresh flower material was poured with boiling water (95 mL, 100 °C) and stirred for 5 min. Then, the infusions were filtered and cooled. In the case of dried flower samples, the same procedure was applied. The weight of dried flower samples was recalculated, basing on dry matter of the material, to obtain 5% infusions imitating extracts from fresh raw material.

### 2.2. Methods

#### 2.2.1. Total Polyphenol Content (TPC)

Total polyphenol content was determined by the method of Singleton et al. [20]. Half of 1 mL of the 5% extract was mixed with 2.5 mL of Folin–Ciocalteu reagent and after 3 min, 5 mL of sodium carbonate solution (75 g/L) was added to the 50 mL volumetric flask. Afterward, the flask was made up to volume with distilled water, the contents mixed and incubated for 2 h at room temperature in darkness. After this time, the absorbance was measured at 750 nm (Cecil CE 9500 spectrophotometer, Cambridge, UK) against a blank, in which the extract was replaced with ethanol. The total polyphenol content was expressed in mg of gallic acid per 100 g of fresh raw material. Analyses were performed in triplicate.

#### 2.2.2. Monomeric Anthocyanins Content (MAC)

The total content of monomeric anthocyanins was determined by differential pH measurement [21]. The volume of 5 mL of the extract was taken into 50 mL volumetric flask and made up to volume with pH 1 buffer (0.025 M KCl + HCl) (1 series) and the second series with 0.4 M acetate buffer at pH 4.5. The samples were incubated for 30 min at room temperature in the dark. The absorbance at 510 nm and 700 nm was then measured on a Cecil CE 9500 spectrophotometer (Cambridge, UK) against distilled water. Measurements were performed in three replicates.

(1) $$MAC\left[\frac{\mathrm{mg}}{L}\right]=\frac{A\times MW\times DF}{e\times l}\cdot 1000$$

where *A*—difference in absorbance:

(2) $$A={\left(A_{510}-A_{700}\right)}_{pH1.0}-{\left(A_{510}-A_{700}\right)}_{pH4.5}$$

*MW* (molecular weight) = 449.2 for cyanidin-3-glucoside,
*DF*—dilution factor = 10,
*e*—molar extinction coefficient = 26.900 for cyanidin-3-glucoside,
*l*—optical path length (*l* = 1 cm).

The results were expressed as mg of cyanidin-3-glucoside per 100 g of fresh material.

#### 2.2.3. Antioxidant Activity Analyses

Antioxidant activity of extracts and infusions was determined by means of four different spectrophotometric techniques. Two radical-scavenging methods were employed: with the use of 2,2-diphenyl-1-picryl hydrazyl (DPPH) according to Brand-William’s method [22] and applying 2,2′-azino-bis(3-ethylobenzotiazolino-6-sulfonate) (ABTS) in accordance with Re et al. [23]. The two other analyses were based on the reduction of Fe^3+^ ions to Fe^2+^ ions, which resulted in intense blue color. In the Benzie and Strain method [24], the ions are complexed by 2,4,6-tris (2-pyridyl)-1,3,5-triazine (TPTZ). In the Yen and Chen method [25], the solution of prussian blue, forming during reduction reaction, is responsible for the sample color. All determinations were performed in three replicates.

##### Determination of Antioxidant Activity Using DPPH Free Radicals

The volume of 0.1 mL of the extract or infusion and 3.9 mL of DPPH solution (0.0236 g/L ethanol) were mixed. The absorbance was measured immediately after mixing at 517 nm (Cecil CE 9500 spectrophotometer, Cambridge, UK) relative to pure ethanol. The next measurement was recorded after one hour. The amount of DPPH free radicals remaining in the reaction mixture after 60 min was calculated as

(3) $$DPPH[\%]=\frac{{\left(A_{517}\right)}_{T0}-{\left(A_{517}\right)}_{T}}{{\left(A_{517}\right)}_{T0}}\cdot 100$$

where [*A*_517_]*_T_*—mixture absorbance at the end of the experiment, [*A*_517_]*_T_*_0_—the absorbance of the mixture at the beginning of the experiment.

##### Determination of Antioxidant Activity Using ABTS Free Radicals

The ABTS radical cations were previously prepared from the 2,2′-azino-bis (3-ethylbenzothiazoline-6-sulfonic acid ammonium salt) by oxidation with sodium persulfate. For this purpose, a stock solution was prepared by dissolving 0.0960 g ABTS and 0.0165 g of sodium persulfate in 25 mL of distilled water. After 24 h, 1 mL was taken from the stock solution and dissolved in 50 mL of PBS (phosphate buffer) solution. The assay was performed by taking 2 mL of the initial solution and 1 mL of extract. The whole mixture was stirred, and after 10 min, the absorbance was measured at 734 nm (Cecil CE 9500 spectrophotometer, Cambridge, UK) against a PBS solution. A reference test was also carried out, measured directly after the addition of free radicals, in which the extract was replaced with a PBS solution. The rate of reduction in the radicals (% RSA) was calculated according to the formula

(4) $$RSA[\%]=\frac{E_{0}-E_{10}}{E_{0}}\cdot 100$$

where

*E*_0_—absorbance of the reaction mixture at the beginning of the analysis,
*E*_10_—absorbance of the reaction mixture at the beginning of the determination.

##### Determination of Antioxidant Activity by the FRAP Method

The working reaction mixture was obtained by mixing an acetate buffer (300 mM, pH 3.6), FeCl_3_ (20 mM) and a TPTZ solution (8 mM) in hydrochloric acid (40 mM) in a 10:1:1 ratio. An amount of 0.4 mL of the extract and 3.6 mL of the TPTZ working solution were mixed and incubated for 10 min at 37°C. Afterward, the solution was centrifuged for 2 min at 4000 rpm. The absorbance of supernatant was measured at 595 nm (Cecil CE 9500 spectrophotometer, Cambridge, England) against a blank, in which the extract was replaced with distilled water. The results were referenced to the standard curve of the Fe^2+^ solution and expressed in μmol/mL of the extract.

##### Determination of Reducing Force

The volume of 1 mL of the extract, 2.5 mL of phosphate buffer (0.2 M, pH 6.6) and 2.5 mL of potassium ferricyanide (K_3_[Fe (CN)_6_]; 10 g/L) were mixed. Afterward, the mixture was incubated at 50 °C for 30 min. Then, 2.5 mL of the trichloroacetic acid solution (100 g/L) was added, mixed, and the whole mixture was centrifuged at 4500 rpm for 15 min. The supernatant (2.5 mL) was transferred to the tube, and 2.5 mL of distilled water together with 0.5 mL of iron (III) chloride solution (1 g/mL) were added. Then, after 7 min, the absorbance was measured at 700 nm (Cecil CE 9500 spectrophotometer, Cambridge, UK), in relation to the blank, in which the extract was replaced with distilled water. The results were recalculated into ascorbic acid equivalents (mg/g), based on standard curve.

#### 2.2.4. Statistical Analysis

All of the quantitative data (TPC [mg/100 g]—total polyphenol content, MAC [mg/100 g]—monomeric anthocyanins content, DPPH [%], ABTS [% RSA]; FRAP [μmol Fe^2+^/mL], RP [mg/g]—reducing power) is presented as the mean value ± standard deviation (SD).

Three-way analysis of variance (ANOVA) was used to check if the analyzed parameters (independent variables) had an influence on the dependent variables [26,27,28]. ANOVA was conducted for each of the following dependent variables: TPC, MAC, DPPH, ABTS, FRAP and RP. The intergroup factors (independent variables) were plants (three levels: red clover, sweet violet, elderberry), type of drying (four levels: fresh, freeze dried, air dried at 30 °C, air dried at 50 °C) and extract (two levels: ethanolic and aqueous).

In case the null hypothesis is rejected based on the ANOVA results (no significant differences between the groups), post hoc tests (multiple comparison tests) must be performed. In order to determine homogeneous groups, the honest significant difference (HSD) of the Tukey’s test was used [26].

Data analysis was performed using Statistica (StatSoft, Inc. 2011 Inc., Tulsa, OK, USA). Values of *p* < 0.05 were considered statistically significant.

The Pearson’s correlation coefficients were also counted for the analyzed parameters (Microsoft Office Excel 2003).

## 3. Results and Discussion

**Figure 1:**
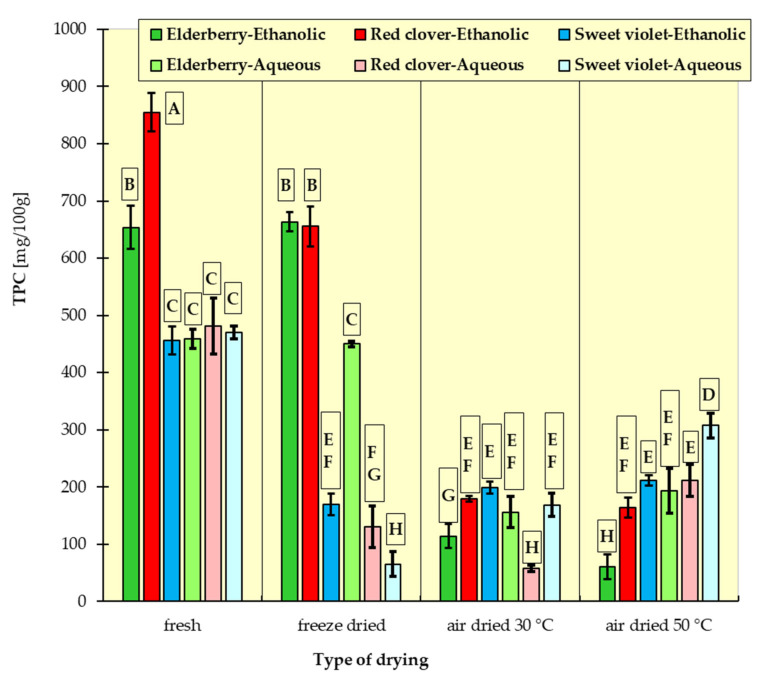
Total polyphenol content (TPC) in ethanolic and aqueous extracts from red clover, sweet violet and elderberry flowers. All data are expressed as mean ± SD. Bars with a different letter indicate significant differences according to HDS Tukey’s test (*p* < 0.05 was accepted as statistically significant). Homogeneous groups are marked with the same letters.

**Figure 2:**
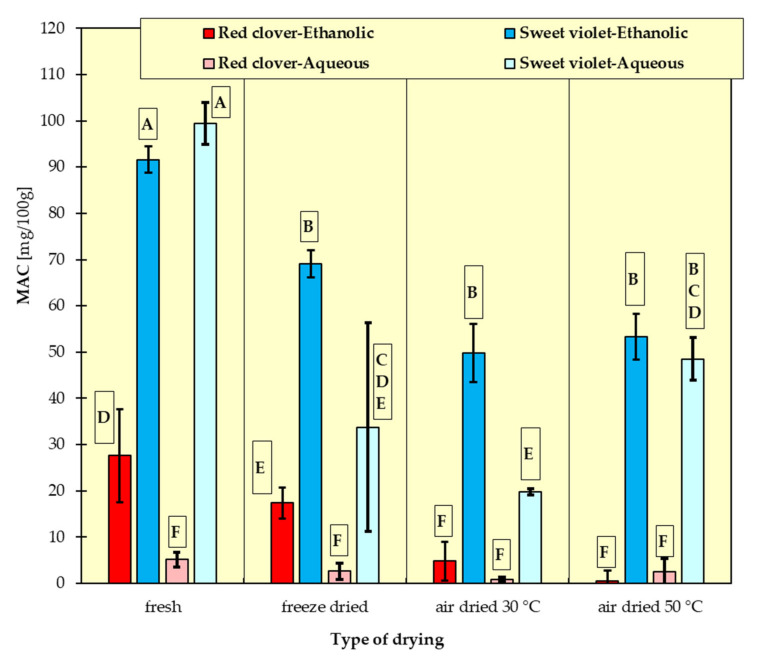
Anthocyanin content (MAC) in ethanolic and aqueous extracts from red clover, sweet violet and elderberry flowers. All data are expressed as mean ± SD. Bars with a different letter indicate significant differences according to HDS Tukey’s test (*p* < 0.05 was accepted as statistically significant). Homogeneous groups are marked with the same letters.

**Figure 3:**
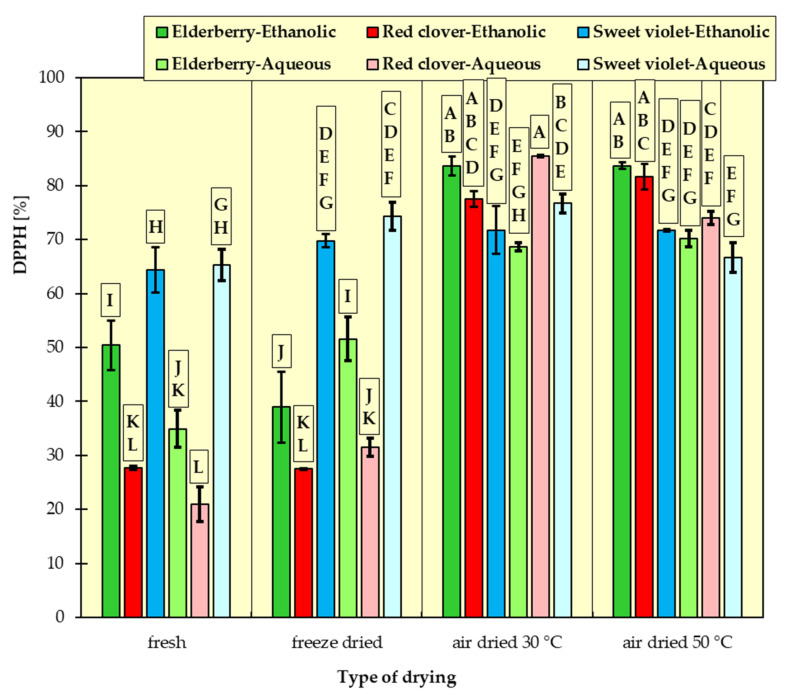
Amount of remaining 2,2-diphenyl-1-picrylhydrazyl (DPPH) as an antioxidant activity measure of ethanolic and aqueous extracts from red clover, sweet violet and elderberry flowers. All data are expressed as mean ± SD. Bars with a different letter indicate significant differences according to HDS Tukey’s test (*p* < 0.05 was accepted as statistically significant). Homogeneous groups are marked with the same letters.

**Figure 4:**
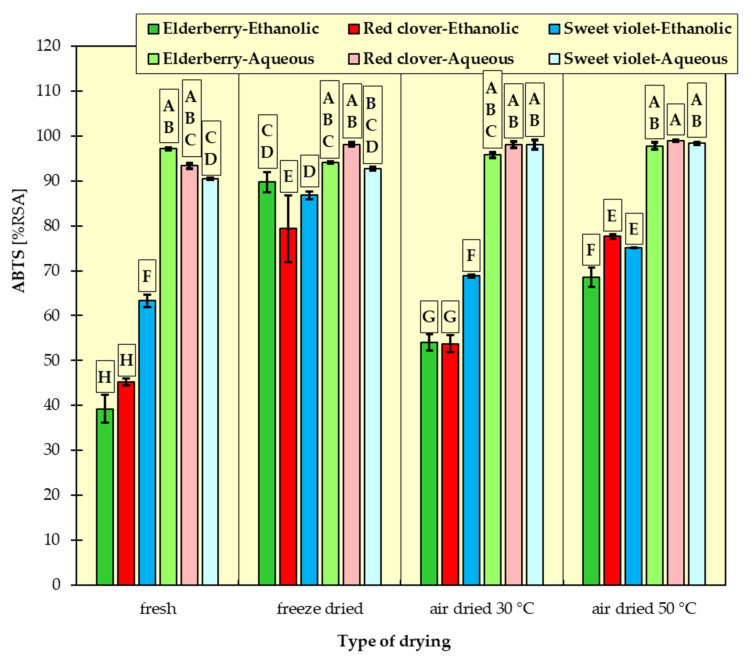
Radical-scavenging activity of ethanolic and aqueous extracts from red clover, sweet violet and elderberry flowers assessed in ABTS method. All data are expressed as mean ± SD. Bars with a different letter indicate significant differences according to HDS Tukey’s test (*p* < 0.05 was accepted as statistically significant). Homogeneous groups are marked with the same letters.

**Figure 5:**
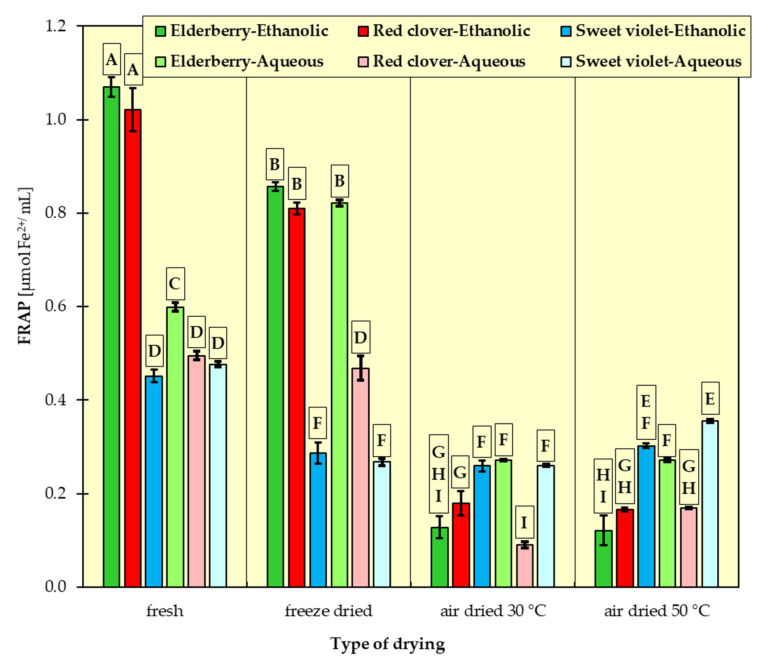
Antioxidant activity of ethanolic and aqueous extracts from red clover, sweet violet and elderberry flowers assessed in FRAP method. All data are expressed as mean ± SD. Bars with a different letter indicate significant differences according to HDS Tukey’s test (*p* < 0.05 was accepted as statistically significant). Homogeneous groups are marked with the same letters.

**Figure 6:**
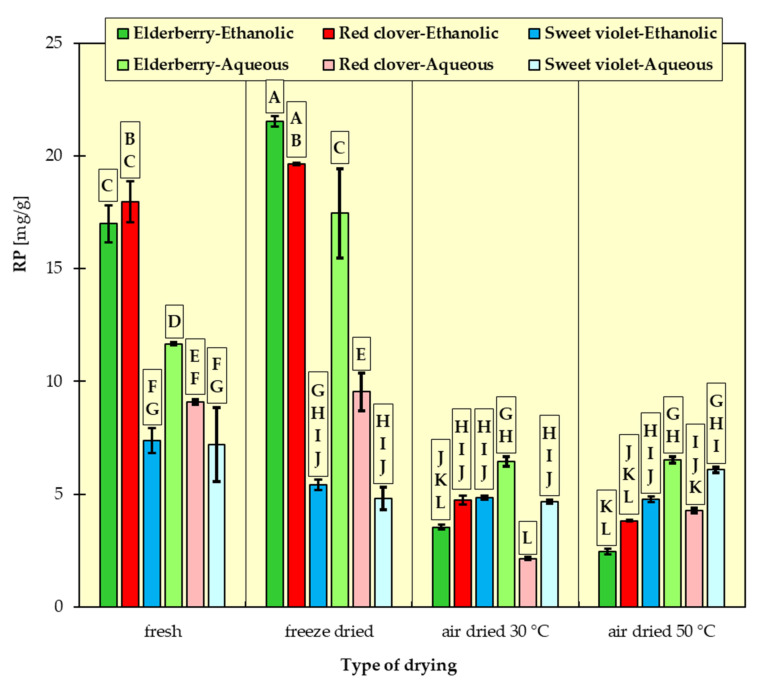
Reducing power of ethanolic and aqueous extracts from red clover, sweet violet and elderberry flowers. All data are expressed as mean ± SD. Bars with a different letter indicate significant differences according to HDS Tukey’s test (*p* < 0.05 was accepted as statistically significant). Homogeneous groups are marked with the same letters.

In Figure 1, Figure 2, Figure 3, Figure 4, Figure 5 and Figure 6, the measurement results for all dependent variables (TPC, MAC, DPPH, ABTS, FRAP and RP) are presented as mean values from three independent measurements ± standard deviation (SD).

### 3.1. Total Polyphenol Content (TPC)

In the group of fresh flowers, the highest content of polyphenols was determined in red clover (855 mg/100 g), a lower content in elderflowers (654 mg/100 g), while the lowest amount was found in sweet violet (456 mg/100 g) (Figure 1).

Generally, a greater amount of polyphenols was determined in ethanolic extracts than aqueous ones, with the exception of sweet violet extracts, with similar content of these compounds in both extracts. This phenomenon can be caused by different qualitative composition within this group of compounds in an individual flower and their different affinity for the solvents used. Edible flowers are a rich source of polyphenols, which vary in quantitative and qualitative contents [1,2,3]. Mikulic-Petkovesk et al. [14] found 1022 mg GAE/100 g of elderflowers, which is 16% more than observed in this work, while Demasi et al. [29] determined only 508 mg GAE per 100 g of fresh *Sambuci flos*. TPC in sweet violet flowers was at the level of 428 mg/100 g [29], which is very similar to what was observed in this study.

Drying, regardless of its type, caused a reduction in the polyphenol content in most samples. The highest stability was demonstrated by phenolic compounds during freeze drying of elderflowers (determined in both ethanolic and aqueous extracts), where no reduction in their quantity was noted compared to fresh samples. Biernacka et al. [30] confirm that the use of freeze drying allows for better preservation of phenolic compounds in the raw material compared to the raw material dried with the use of warm air. In addition, the TPC values obtained by Viapiana and Wesołowski [15] for various elderflower infusions prepared from commercially available dried products were quite similar to those determined in this work. There are, however, scientific reports informing about higher content of these compounds in *Sambuci flos* [31,32].

The largest decrease in the content of polyphenols in lyophilized samples was recorded for sweet violet flowers (aqueous extract). In turn, air drying at 30 °C and 50 °C led to losses in the content of polyphenols in elderflower ethanolic extracts by 82.5% and up to 91%, respectively. Opposite results were found for sweet violet and red clover flowers dried at 50 °C, especially with regard to aqueous extracts, in which the TPC was higher compared to the samples dried at 30 °C. This phenomenon may be due to the fact that these flowers contain a large amount of polyphenol oxidase [33,34], an enzyme that degrades phenolic compounds, and only the application of a drying temperature of 50 °C induces its inactivation and thus reduces the loss of polyphenolic compounds. As described by Cheng at al. [35], PPO enzymes can remain unaffected by mild heat treatments, particularly when the drying temperature is below 55°C [35]. Selvi et al. [36] observed that increasing the temperature and drying time of rose (*Rosa ‘Electron’*) petals resulted in an increase in TPC in this material. According to Stefaniak and Grzeszczuk [37], who studied the effect of drying temperature on, i.a., the content of polyphenols in selected edible flowers, of the three temperatures used (25 °C, 35 °C and 70 °C), in most cases, 35 °C was the most favorable for the retention of these compounds. From a review by Fernandes at al. [38], it can be concluded that the drying method should be individually selected for a given species of edible flower.

The TPC determined in this research in dry sweet violet flowers was larger than that reported by Tünde et al. [39]. In turn, the content of polyphenolic compounds noted by Tava et al. [10] in methanolic extract of freeze-dried *Trifolium pratense* flowers was higher than that observed in lyophilized samples in this work. The content of polyphenols in the methanolic extract of red clover bud phase (dried at 60 °C), stated by Vlaisavljević et al. [8], was higher than in flowers dried at 50 °C in this study. It has to be emphasized that the amount of determined polyphenols depends not only on the drying method but also on the extraction procedure.

### 3.2. Monomeric Anthocyanins Content (MAC)

Anthocyanins were not detected in *Sambuci flos*, which is congruent with the findings of other authors [31,32,40]. Fresh sweet violet flowers had the highest anthocyanin content (93 mg/100 g in ethanolic extract and 98.7 mg/100 g in aqueous extract). Lyophilization allowed the greatest retention of these compounds that were soluble in ethanol, while drying at 50 °C proved to be a good way to preserve the anthocyanin fraction more soluble in water (Figure 2).

Singh et al. [12] reported that dried *Viola odorata* flowers contain 4% of anthocyanins, while Karioti et al. [41] obtained very poor content of these compounds and assigned this to thermal degradation during the sample preparation prior to analysis.

According to Lee et.al [42], *Trifolium pratense* flowers dried at room temperature had more anthocyanins than any sample examined in this study.

### 3.3. Antioxidant Activity Analyses

Antioxidant activity was determined by four methods to make the assessment of the antioxidant capacity more complete.

An analysis of the results (Figure 3, Figure 4, Figure 5 and Figure 6) revealed that fresh red clovers and elderflowers were the flowers with the greatest antioxidant capacity, and freeze drying was the best method. As for the reducing power and the FRAP assays, higher antioxidant activity was found for ethanolic extracts. In turn, in the DPPH method, the choice of the suitable solvent was not clear cut. Only the RSA obtained on the basis of ABTS scavenging method differed from the others. In this case, the aqueous extracts of all the examined flowers had higher free radical quenching capacity compared to the ethanolic extracts. In addition, the highest such ability was found for the aqueous extracts of flowers dried conventionally at 30 °C and 50 °C. High antioxidant potential can be attributed to the relatively high polyphenol content of individual flowers. There are numerous studies confirming this relationship [15,32,43,44], in which it was proved that ethanolic elderflower extracts exhibited strong activity in scavenging DPPH radicals. The antioxidant potential was strongly dependent on the extraction procedure.

### 3.4. Statistical Analysis

The ANOVA results (Table 1) show a statistically significant effect of the independent variables (Plant, Drying and Extract) as well as their interactions (Plant*Drying, Plant*Extract, Drying*Extract and Plant*Drying*Extract) on all six dependent variables (TPC, MAC, DPPH, ABTS, FRAP and RP) apart from Plant*Extract for dependent variable MAC.

The results of Tukey’s HDS test for the dependent variables are presented in Figure 1, Figure 2, Figure 3, Figure 4, Figure 5 and Figure 6, respectively. Homogeneous groups are marked with the same letters. Statistical analysis was performed for *p* < 0.05.

Table 2 presents the Pearson’s correlation coefficients between the values of the analyzed parameters without distinguishing between the results concerning the ethanolic extracts and the aqueous ones, as well as preservation method. This allows a more general view of the obtained results.

When analyzing these data, it can be observed that the results obtained from three methods (DPPH, FRAP, reducing power—RP) of testing antioxidant activity are strongly correlated with each other. The antioxidant activity determined by the ABTS method did not show much correlation with the results obtained in the remaining methods. There is strong correlation between antioxidant properties and TPC, which is consistent with some scientific reports [45,46]. However, there is no such relationship in relation to anthocyanin content. The type of observed correlations depends on the structure of polyphenolic compounds occurring in the tested material.

## 4. Conclusions

Edible flowers are a valuable and, at the same time, very perishable material. So, in order to preserve their precious properties, an appropriate method of their conservation should be developed. This study showed that in most cases, freeze drying preserves well the antioxidant properties of the examined raw materials, as well as the compounds determining these properties. Moreover, the choice of an appropriate solvent for the extraction of bioactive compounds is extremely important to obtain a product with specific features. The selection of a suitable solvent is an individual matter for each edible flower, due to their diverse chemical composition and interactions of chemical compounds in the biological matrix. Of the studied flowers, the highest amount of polyphenols was determined in the ethanolic extracts of fresh red clover flowers (854.76 mg/100 g), while the highest concentration of anthocyanins was detected in the aqueous extracts of fresh sweet violet flowers (99.41 mg/100 g). The flowers of red clover had the highest antioxidant activity among the analyzed edible flowers, followed by the elderflowers and sweet violet. However, these values fluctuated depending on the method used. This research also revealed that polyphenolic compounds were mainly responsible for the antioxidant activity of the studied edible flowers, since antioxidant activity and TPC (r = 0.9196, for FRAP method) were strongly correlated. There was no such relationship in the case of anthocyanins.

## Figures and Tables

**Table 1:** ANOVA results for TPC, MAC, DPPH, ABTS, FRAP and RP in samples of red clover, sweet violet and elderberry flowers.

Intergroup Factors	*p* TPC ^1^	*p* MAC ^2^	*p* DPPH ^3^	*p* ABTS ^4^	*p* FRAP ^5^	*p* RP ^6^
Plant	0.0000 *	0.0000 *	0.0000 *	0.0000 *	0.0000 *	0.0000 *
Drying	0.0000 *	0.0000 *	0.0000 *	0.0000	0.0000 *	0.0000 *
Extract	0.0000 *	0.0000 *	0.0008 *	0.0000 *	0.0000 *	0.0000 *
Plant*Drying	0.0000 *	0.0000 *	0.0000 *	0.0000	0.0000 *	0.0000 *
Plant*Extract	0.0000 *	0.1595	0.0000 *	0.0000	0.0000 *	0.0000 *
Drying*Extract	0.0000 *	0.0012 *	0.0000 *	0.0000	0.0000 *	0.0000 *
Plant*Drying*Extract	0.0000 *	0.0001 *	0.0000 *	0.0000	0.0000 *	0.0000 *

Significant results are indicated by asterisks * (*p* < 0.05). ^1^ TPC [mg/100 g]—total polyphenol content; ^2^ MAC [mg/100 g]—monomeric anthocyanins content; ^3^ DPPH [%]; ^4^ ABTS [% RSA]; ^5^ FRAP [μmol Fe^2+^/mL]; ^6^ RP [mg/g]—reducing power.

**Table 2:** Correlation coefficients between values of analyzed parameters.

Parameters	TPC ^1^	MAC ^2^	DPPH ^3^	ABTS ^4^	FRAP ^5^	RP ^6^
TPC	1	0.2354	−0.7623	−0.3197	0.9196	0.8629
MAC		1	0.0048	−0.1781	0.0994	−0.0051
DPPH			1	−0.0205	−0.7908	−0.8084
ABTS				1	−0.2799	−0.0201
FRAP					1	0.9602
RP						1

^1^ TPC [mg/100 g]—total polyphenol content; ^2^ MAC [mg/100 g]—monomeric anthocyanins content; ^3^ DPPH [%]; ^4^ ABTS [% RSA]; ^5^ FRAP [μmol Fe^2+^/mL]; ^6^ RP [mg/g]—reducing power.

## Data Availability

The data presented in this study are available within the article.
